# A Fusion Localization System for Security Robots Based on Millimeter Wave Radar and Inertial Sensors

**DOI:** 10.3390/s24237551

**Published:** 2024-11-26

**Authors:** Rui Zheng, Geng Sun, Fang Dong Li

**Affiliations:** 1 College of Physics and Electronic Information, Anhui Normal University, Wuhu 241002, China; zrwx0609@ahnu.edu.cn (R.Z.); kai_xuan2233@163.com (G.S.); 2 College of Automation Engineering, Nanjing University of Aeronautics and Astronautics, Nanjing 210016, China

**Keywords:** security robot, Kalman filtering, millimeter wave, localization, inertial sensor

## Abstract

In smoggy and dusty environments, vision- and laser-based localization methods are not able to be used effectively for controlling the movement of a robot. Autonomous operation of a security robot can be achieved in such environments by using millimeter wave (MMW) radar for the localization system. In this study, an approximate center method under a sparse point cloud is proposed, and a security robot localization system based on millimeter wave radar is constructed. To improve the localization accuracy of the robot, inertial localization of the robot is integrated with MMW radar. Based on the concept of inertial localization, the state equation for the motion principle of the robot is deduced. According to principle of MMW localization, the measurement equation is derived, and a kinematics model of the robot is constructed. Further, by applying the Kalman filtering algorithm, a fusion localization system of the robot based on MMWs and inertial localization is proposed. The experimental results show that with iterations of the filtering algorithm, the gain matrix converges gradually, and the error of the fusion localization system decreases, leading to the stable operation of the robot. Compared to the localization system with only MMW radar, the average localization error is approximately reduced from 11 cm to 8 cm, indicating that the fusion localization system has better localization accuracy.

## 1. Introduction

Security robots are intelligent agents that perform dangerous tasks on behalf of humans [1,2] in adverse environments such as smog and dust. To enable the autonomous movement of the robot, the position of the robot must be obtained and its movement must be controlled in real time; therefore, localization technology is a key technology for the robot [3,4]. Laser-based and vision-based localization are common methods for mobile robot localization [5,6]. For instance, the university of Luxembourg [7] utilized Lidar to construct and optimize, in real time, a three layered Situational Graph that included a robot-tracking layer where the robot’s poses are registered, a metric–semantic layer with features such as planar walls, and a novel topological layer constraining the planar walls using higher-level features such as corridors and rooms. This graph can be used for robot localization. Zou et al. [8] analyzed the characteristics and performances of different Lidar simultaneous localizations and mappings and summarized their application to indoor localization. Although cartographic localization accuracy is high, over time, cartographers will consume more and more memory to store newly constructed maps. Another study [9] proposed a maples visual localization system for bipedal humanoid robots. In this method, information is extracted from color images using deep reinforcement learning to derive motion commands. In addition, a robot localization system using a Kinect sensor and the concept of transfer learning based on convolutional neural networks was proposed [10]. These studies indicate that laser and visual localization technologies are widely used and have high precision.

However, in case of working environments such as smog or dust, achieving a precise laser positioning focus point is difficult due to the disturbance of air density [11]. In a dust environment, environmental information cannot be fully collected using visual sensors, thus hindering the application of visual localization methods [12]. To realize effective localization of robots in smog and dust environments, a sensor with strong anti-interference ability needs to be studied. MMWs, with a frequency band of 30–300 GHz, have characteristics of both infrared and microwaves, thus possessing the characteristics of strong anti-interference ability, low power consumption, strong penetration, and high Doppler frequency [13]. Therefore, the application of MMW radar in smog and dust environments can be used for the localization of robots in such environments [14]. The results indicated that the resolution of detection targets using MMW is better than that using optical sensors. Laser localization cannot be used for robots in a mine environment, according to the analysis in [15]; hence, in that study, the technology of simultaneous location and mapping using MMWs was proposed to realize the autonomous localization of robots. The triangulation method, based on frequency-modulated MMW radar, was first proposed in [16]. By scanning the robot with an MMW radar, the distribution information of point clouds was obtained; further, a cut-clustering method was used to effectively reduce the ranging error and localization error caused by the volume of the robot. Based on that method, the security robot localization system was designed, which was experimentally confirmed to realize autonomous operation of the robot in smog and dust environments, with a localization error of approximately 0.11 m.

While performing dangerous tasks, the robot is usually required to reach the target point as accurately as possible [17]. For example, the robot needs to be accurately moved to a specific position during the task of cleaning of dangerous and flammable objects [18]. One of the effective ways to improve the working efficiency of the robot is to improve its localization accuracy. Combining MMW radar and other sensors is an effective method to improve localization accuracy [19]. Inertial and other localization methods are commonly combined with MMWs. For example, Li et al. [20] proposed an architecture based on the recurrent convolutional neural network, which combines laser and inertial data to achieve high-precision positioning of the robot. An integrated autonomous relative localization method based on vision and inertial measurement unit (IMU) data fusion was proposed in [21]. The experimental results showed that this method has high accuracy and robustness. A localization system based on low-cost vision and inertial fusion and using the extended Kalman filter (EKF) algorithm was proposed in [22]; experiments revealed a localization accuracy of approximately 0.3 m. In general, inertial sensors are not affected by external environments and have strong anti-interference ability and good localization accuracy within a short time [23]. Therefore, inertial sensors can also be used in smog and dust environments. A localization method combining frequency-modulated continuous-wave (FMCW) radar and an inertial odometer was proposed in [24]. FMCW radar is fixed on the carrier, and its localization error increases with the increase in the robot’s moving distance. In [25,26], a depth fusion method of MMW attitude estimation and other sensors was proposed to accurately estimate the motion trajectory of the carrier; in this method also, the MMW radar is fixed to the carrier.

From the above analysis, it can be seen that millimeter wave radar localization systems can output the localization information of security robots in smog and dust environments. Inertial sensors can provide continuous localization and attitude information for robots, with high short-term accuracy. The millimeter wave radar localization system has stable localization accuracy, but its continuous localization capability is inferior to that of inertial sensors. Therefore, this paper focuses on further improving the localization accuracy of security robots in smog and dust environments, and carries out the research based on the fusion of millimeter wave and inertial sensors. In this study, the MMW radar is placed in the carrier motion space, the position of the robot is measured by the triangular positioning method, and the positioning accuracy is improved by incorporating inertial sensor information. In this paper, based on inertial localization theory and frequency-modulated MMW localization theory, the state equation and the measurement equation of the robot are constructed. Then, the Kalman algorithm is used. A comparative study on the filtering algorithm is designed, and the fusion localization method is proposed. Autonomous localization of the robot in smog and dust environments is achieved with high accuracy. This paper’s contributions are summarized as follows:

(1) In order to obtain the approximate geometric center of the target under the sparse point cloud, based on the point cloud information of the millimeter wave radar, an approximate center method based on the sparse point cloud is proposed.
(2) Because of the localization requirements of indoor security robots, inertial localization systems can provide continuous localization and attitude information. And the inertial localization system has pure autonomy, and can be well adapted to a smog environment. Therefore, on the basis of the previous research of our group, in order to further improve the continuous localization capabilities of security robots in smog and dark environments, and to enhance the accuracy of continuous localization of security robots, this paper proposes a method of fusing an inertial localization system with a millimeter wave localization system. While improving the localization accuracy of the security robot, it also enhances the continuous localization ability of the security robot.
(3) A hardware platform for the security robot based on millimeter wave and inertial localization fusion is designed.

## 2. Design of Localization System

Prior to establishing the mathematical model of the fusion localization system, it is necessary to study the basic principles of the MMW localization system and inertial localization system (INS).

### 2.1. MMW Localization System

The basic principle of the MMW localization system is based on the distance between the MMW radars and the robot, obtained using triangulation to calculate and control the position of the robot.

After normalizing the signal amplitude of a millimeter wave radar, the transmitted signal and the echo signal reflected back at the distance *d* are:

(1) $$\left\{\begin{array}{c} X_{\mathrm{trans}}\left(t\right)=\cos\left(2\pi f_{0}t+\pi \mu t^{2}\right)\\ X_{\mathrm{rec}}\left(t\right)=\cos\left[2\pi f_{0}\left(t-\Delta \tau \right)+\pi \mu {\left(t-\Delta \tau \right)}^{2}\right] \end{array}\right.$$

In the formula, $f_{0}\;$ is the starting frequency of the millimeter wave radar’s frequency-modulated continuous wave. μ is the modulation slope. ∆*t* = 2d/c is the time delay. c is the speed of light. d is the distance between the measured object and the MMW radar. The complex signal $X_{mix}(t)$ obtained by mixing the received signal with the transmitted signal is:

(2) $$X_{\mathrm{mix}}\left(t\right)=\exp[\frac{j\left(4\pi \mu dt\right)}{c}+j\left(\frac{2d}{c}\right)\left(2\pi f_{0}-\frac{2\pi \mu d}{c}\right)]$$

The instantaneous frequency $f_{i}$ of the signal is:

(3) $$f_{i}=\frac{1}{2\pi }\frac{d[\frac{4\pi \mu dt}{c}+\frac{2d}{c}(2\pi f_{0}-\frac{2\pi \mu d}{c})]}{dt}=\frac{2\mu d}{c}$$

According to Formula (3), the instantaneous frequency is proportional to the distance. By sampling the echo signal and using fast Fourier transform processing, the target distance corresponding to the peak position of $f_{i}$ is:

(4) $$d=f_{i}cT_{m}/2\Delta F$$

where ΔF is the bandwidth of the modulated signal and $T_{m}$ is the modulation period.

For the i-th signal source received by the first array element of the radar M array elements, it can be obtained through Nyquist sampling:

(5) $$\begin{array}{l} \;\;X_{l}\left(t\right)=\sum_{i=1}^{N}A_{T}A_{R}S_{i}\left(t-\tau_{li}\right)+N_{l}\left(t\right)\\ \;l=1,2,\cdots ,M\begin{array}{cc} & \;i=1,2,\cdots ,N \end{array} \end{array}$$

where $X_{l}\left(t\right)$ is the ADC sampling frequency of the l-th signal of $X\left(t\right)$. $A_{T}A_{R}$ is the gain of the first array element to the i-th signal, and $N_{l}\left(t\right)$ is the additive noise of the first array element. $S_{i}\left(t-\tau_{li}\right)$ refers to the signal from the i-th signal to the lth array element under the delay of $\tau_{li}$. The expression of delay is:

(6) $$\tau_{ki}=\left(d_{k}\sin\theta_{i}\right)/c\begin{array}{cc} & k=1,\;2,\cdots ,l,\;M \end{array}$$

where $d_{k}$ is the position of the array element, and $\theta_{i}$ is the azimuth of a signal source and the radar. According to Formulas (3) and (4), the $\sum_{i=1}^{N}A_{T}A_{R}S_{i}\left(t-\tau_{ki}\right)$ of M array elements is simplified as follows:

(7) $$\sum_{i=1}^{N}A_{T}A_{R}S_{i}\left(t-\tau_{ki}\right)\approx \sum_{i=1}^{N}A_{T}A_{R}S_{i}\left(t\right)\exp\left(-j\omega_{0}\tau_{ki}\right)$$

where $\omega_{0}$ is the frequency of the received signal. Let the $\exp\left(-j\omega_{0}\tau_{ki}\right)$ of Formula (5) be transformed to obtain:

(8) $$\alpha \left(\theta_{i}\right)=\exp\left(-j\omega_{0}\tau_{Mi}\right)=\exp\left(-j\omega_{0}x_{k}\sin\theta_{i}/c\right)$$

Utilizing the Capon algorithm:

(9) $$P\left(\theta \right)=1/\left[\alpha^{H}\left(\theta \right)R^{-1}\alpha \left(\theta \right)\right]$$

where $R=X\left(t\right)\cdot X^{H}\left(t\right)/L$, and *L* is the number of snapshots. $P\left(\theta \right)$ is the set spectral range, which is from positive 90 degrees to negative 90 degrees. Search the spectral peak of one dimension of $P\left(\theta \right)$, such that the abscissa corresponding to the peak is the azimuth θ, so as to obtain the azimuth of the signal.

Based on the expression measuring the quality of radar received signal, we have:

(10) $$\mathrm{SNR}=\frac{\sigma P_{t}G_{\mathrm{TX}}G_{\mathrm{RX}}\lambda^{2}T_{\mathrm{meas}}}{{\left(4\pi \right)}^{3}d^{4}K_{m}TF}$$

where SNR is the signal-to-noise ratio. σ is the scattering cross-section. $P_{t}$ is the radar output power. $G_{\mathrm{TX}}$ and $G_{\mathrm{RX}}$ are the antenna receiving and transmitting power. λ is the signal wavelength. $T_{\mathrm{meas}}$ is the pulse modulation time. d is the distance between the radar and the target. T is the temperature. F is the radar internal noise coefficient, and $K_{m}$ is the antenna noise coefficient.

**Figure 1:**
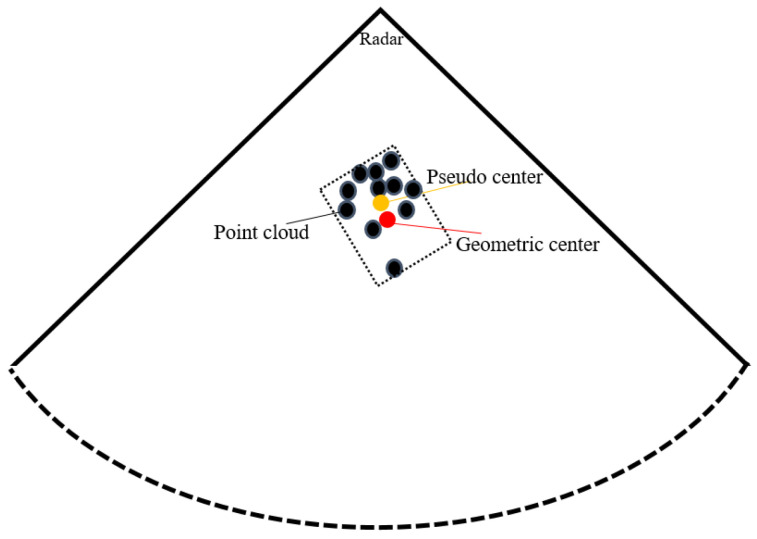
Radar point cloud distribution (red is the geometric center of the target, and yellow is the point cloud information after mean processing).

According to Formula (10), the signal-to-noise ratio of the processed signal is inversely proportional to the fourth power of the distance and is proportional to the radar cross-section. Therefore, the number of target point clouds will be unevenly distributed due to the distance between the reflector and the radar. If the mean value of these point clouds is directly processed, the geometric center distance between the radar and the target will be offset, as shown by the yellow dots in Figure 1.

For this kind of situation, this paper proposes an approximate center method under a sparse point cloud. The set of radial distance between the point cloud and radar is:

(11) $$d=\left[d_{1},d_{2},\cdots ,d_{M}\right]$$

The set of angles between the point cloud and radar is:

(12) $$\theta =\left[\theta_{1},\theta_{2},\cdots ,\theta_{M}\right]$$

The sets of the point cloud between the radar and robot are:

(13) $$p\left(d,\theta \right)=\left[p_{1}\left(d_{1},\theta_{1}\right),p_{2}\left(d_{2},\theta_{2}\right),\cdots ,p_{M}\left(d_{M},\theta_{M}\right)\right]$$

Assume that the maximum angle range of the radar is ϖ, and the maximum detection range is l, and divide Figure 1:

(14) $$\left\{\begin{array}{l} \varpi_{m-1}-\varpi_{1}=\left(m-1\right)\Delta \varpi \\ l_{e-1}-l_{1}=\left(e-1\right)\Delta l \end{array}\right.$$

where $\varpi_{1}$ is the starting angle scale value. $\varpi_{M-1}$ is the ending angle scale value. m−1 is the division multiple, and Δϖ is the angle division interval. $l_{1}$ is the starting distance scale. $l_{M-1}$ is the ending distance scale value. Δl is the distance division interval, and e−1 is the division multiple. Its composition range is:

(15) $$K_{me}=\left(m-1\right)\times \left(e-1\right)$$

According to the distribution of in, it can be divided into G parts, and its expression is:

(16) $$\left\{\begin{array}{c} G\left(M_{m},D_{d}\right)=\left[G_{1}\left(d,\theta \right),G_{2}\left(d,\theta \right),\cdots ,G_{m}\left(d,\theta \right)\right]\\ G_{1}\left(d,\theta \right)\cup G_{2}\left(d,\theta \right)\cup \cdots \cup G_{m}\left(d,\theta \right)=p\left(d,\theta \right)\\ G\left(M_{m},D_{d}\right)\subseteq K_{me}\left(m,e\right) \end{array}\right.$$

where $G_{1}\left(d,\theta \right),G_{2}\left(d,\theta \right),\cdots ,G_{m}\left(d,\theta \right)$ represents all subsets in the $G\left(M_{m},D_{d}\right)$ region, and the set elements of Formula (10) are distributed in the G region. After calculating the mean value of each subset of the G region, we can obtain:

(17) $$d_{p}=\frac{E\left[G_{1}\left(d,\theta \right)\right]+E\left[G_{2}\left(d,\theta \right)\right]+\cdots +E\left[G_{m}\left(d,\theta \right)\right]}{G}$$

According to Formula (17), the approximate geometric center distance between the radar and the target can be obtained.

**Figure 2:**
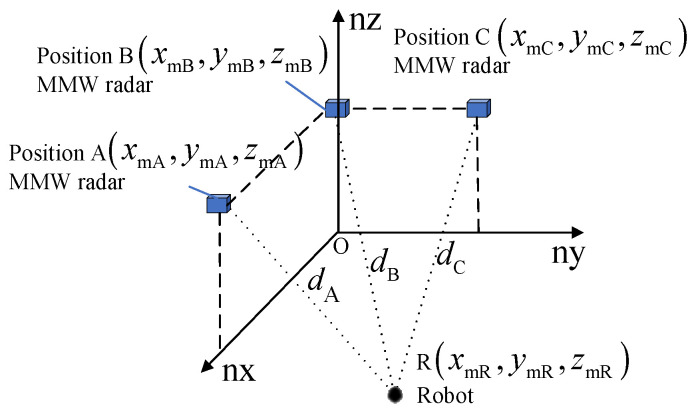
Millimeter wave triangulation localization scheme.

Then, three radars are arranged to form the scheme of triangulation, as shown in Figure 2.

As shown in Figure 2, three radars are placed at points A, B, and C in the localization coordinate system, and their coordinates are $\left(x_{\mathrm{mA}},\;y_{\mathrm{mA}},\;z_{\mathrm{mA}}\right)$, $\left(x_{\mathrm{mB}},\;y_{\mathrm{mB}},\;z_{\mathrm{mB}}\right)$, and $\left(x_{\mathrm{mC}},\;y_{\mathrm{mC}},\;z_{\mathrm{mC}}\right)$, respectively. The distances from points A, B, and C to the robot are $d_{A}$, $d_{B}$, and $d_{C}$, respectively. R $\left(x_{\mathrm{mR}},\;y_{\mathrm{mR}},\;z_{\mathrm{mR}}\right)$, based on the basic principles of triangulation, can then be obtained.

### 2.2. Strapdown Inertial Localization System

In the strapdown INS, the gyroscope and accelerometer are fixed on the robot, with their axes consistent with the carrier body coordinate system; the x-axis is aligned with the direction of the robot’s movement; the direction of the z-axis is perpendicular to the ground; and the direction of the y-axis is determined by the right-hand rule. Using the gyroscope, the attitude matrix of the robot is obtained by measuring the angular motion information. The acceleration of the robot in the localization coordinate system can be expressed as:

(18) $$\left[\begin{array}{c} a_{\mathrm{nx}}\\ a_{\mathrm{ny}}\\ a_{\mathrm{nz}} \end{array}\right]=\left[\begin{array}{ccc} \cos\beta \cos\psi & \cos\beta \sin\psi & -\sin\beta \\ \sin\gamma \sin\beta \cos\psi -\cos\gamma \sin\psi & \sin\gamma \sin\beta \cos\psi +\cos\gamma \cos\psi & \sin\gamma \cos\beta \\ \cos\gamma \sin\beta \cos\psi +\sin\gamma \sin\psi & \cos\gamma \sin\beta \sin\psi -\sin\gamma \cos\psi & \cos\gamma \cos\beta \end{array}\right]\left[\begin{array}{c} a_{\mathrm{bx}}\\ a_{\mathrm{by}}\\ a_{\mathrm{bz}} \end{array}\right]-\left[\begin{array}{c} 0\\ 0\\ g \end{array}\right]$$

where g is the gravity acceleration, and γ, β, and ψ are the rotation angles of the robot along the x, y, and z axes, respectively. ${\left[\begin{array}{ccc} a_{\mathrm{bx}} & a_{\mathrm{by}} & a_{\mathrm{bz}} \end{array}\right]}^{T}$ is the acceleration of the robot in the carrier body coordinate system, while ${\left[\begin{array}{ccc} a_{\mathrm{nx}} & a_{\mathrm{ny}} & a_{\mathrm{nz}} \end{array}\right]}^{T}$ is the acceleration of the robot in the localization coordinate system. To combine the two localization systems, the inertial localization coordinate system is consistent with the MMW localization coordinate system.

The velocity of the robot can be obtained by integrating ${\left[\begin{array}{ccc} a_{\mathrm{nx}} & a_{\mathrm{ny}} & a_{\mathrm{nz}} \end{array}\right]}^{T}$, and the position of the robot can be obtained by integrating the velocity. Due to the noise in ${\left[\begin{array}{ccc} a_{\mathrm{bx}} & a_{\mathrm{by}} & a_{\mathrm{bz}} \end{array}\right]}^{T}$, the velocity and position data of the robot are reliable for a short time, and the errors in the data are divergent with increasing time.

## 3. Fusion Localization System

An MMW localization system can work in smog and dust environments and has the characteristic of nondivergence of positioning errors. A strapdown INS has good short-term accuracy, can be integrated with the MMW localization system to improve the positioning accuracy of the robot, and can provide localization data for the system in a short time when the MMW signal is blocked.

To realize the fusion of the MMW and the INS, it is necessary to study the state equation and measurement equation, thereby constructing a mathematical model of the fusion system.

### 3.1. State Equation

In the strapdown INS, T is the sampling time, and Δv is the velocity variation, which is detected using an accelerometer. $\Delta v_{k}$ indicates the difference in velocity at the (k − 1)-th and k-th moments. From k − 1 to k, the average value of acceleration ${ā}_{n(k-1)}$ in localization coordinate is:

(19) $${ā}_{n(k-1)}=\Delta v_{k}/T$$

${ā}_{\mathrm{nx}(k-1)}$, ${ā}_{\mathrm{ny}(k-1)}$, and ${ā}_{\mathrm{nz}(k-1)}$ represent the average acceleration of the robot along the $n_{x}$, and $n_{y}$ axes, respectively. The position of the robot can be expressed as:

(20) $$\left\{\begin{array}{c} x_{R(k)}=x_{R(k-1)}+v_{x(k-1)}T+1/2T^{2}{ā}_{\mathrm{nx}(k-1)}\\ y_{R(k)}=y_{R(k-1)}+v_{y(k-1)}T+1/2T^{2}{ā}_{\mathrm{ny}(k-1)}\\ z_{R(k)}=z_{R(k-1)}+v_{z(k-1)}T+1/2T^{2}{ā}_{\mathrm{nz}(k-1)} \end{array}\right.$$

where $x_{R(k)}$, $y_{R(k)}$, and $z_{R(k)}$ represent the position of the robot along the $n_{x}$, $n_{y}$, and $n_{z}$ axes at time k. $x_{R(k-1)}$, $y_{R(k-1)}$, and $z_{R(k-1)}$ represent the position of the robot along the $n_{x}$, $n_{y}$, and $n_{z}$ axes at time k − 1. $v_{x(k)}$, $v_{y(k)}$, and $v_{z(k)}$ represent the velocity of the robot along the $n_{x}$, $n_{y}$, and $n_{z}$ axes at time k, which are obtained through the Doppler effect of millimeter wave radar and coherent measurement methods. Let ${ā}_{\mathrm{nx}(k-1)}$, ${ā}_{\mathrm{ny}(k-1)}$, and ${ā}_{\mathrm{nz}(k-1)}$ represent the true values of the average accelerations; then, Equation (20) can be expressed as:

(21) $$\left[\begin{array}{c} x_{R(k)}\\ y_{R(k)}\\ z_{R(k)} \end{array}\right]=\left[\begin{array}{ccc} 1 & 0 & 0\\ 0 & 1 & 0\\ 0 & 0 & 1 \end{array}\right]\left[\begin{array}{c} x_{R(k-1)}\\ y_{R(k-1)}\\ z_{R(k-1)} \end{array}\right]+\left[\begin{array}{cccccc} T^{2}/2 & 0 & 0 & T & 0 & 0\\ 0 & T^{2}/2 & 0 & 0 & T & 0\\ 0 & 0 & T^{2}/2 & 0 & 0 & T \end{array}\right]\left[\begin{array}{c} {{ā}^{’}}_{\mathrm{nx}(k-1)}\\ {{ā}^{’}}_{\mathrm{ny}(k-1)}\\ {{ā}^{’}}_{\mathrm{nz}(k-1)}\\ v_{x(k-1)}\\ v_{y(k-1)}\\ v_{z(k-1)} \end{array}\right]+\left[\begin{array}{ccc} 1 & 0 & 0\\ 0 & 1 & 0\\ 0 & 0 & 1 \end{array}\right]\left[\begin{array}{c} w_{x\left(k-1\right)}\\ w_{y\left(k-1\right)}\\ w_{z\left(k-1\right)} \end{array}\right]$$

where $w_{x\left(k-1\right)}$, $w_{y\left(k-1\right)}$, and $w_{z\left(k-1\right)}$ represent the noise of the accelerometer along the $n_{x}$, $n_{y}$, and $n_{z}$ axes from k − 1 to k.

In the localization system, the position coordinate of the robot is an important state quantity. $R_{k}={\left[\begin{array}{ccc} x_{R\left(k\right)} & y_{R\left(k\right)} & z_{R\left(k\right)} \end{array}\right]}^{T}$ is a state quantity at k, and $R_{k-1}={\left[\begin{array}{ccc} x_{R\left(k-1\right)} & y_{R\left(k-1\right)} & z_{R\left(k-1\right)} \end{array}\right]}^{T}$ is a state quantity at k−1. The discrete-time state equation is shown in the following equation:

(22) $$R_{k}=\Phi R_{k-1}+\Lambda {\bar{V}}_{k-1}+ϒw_{k-1}$$

where $\Phi =\mathrm{diag}\left[\begin{array}{ccc} 1 & 1 & 1 \end{array}\right]$ is the state transition matrix. $\Lambda =diag\left[\begin{array}{ccc} \Delta T & \Delta T & \Delta T \end{array}\right]$ is the control transition matrix. $ϒ=\mathrm{diag}\left[\begin{array}{ccc} 1 & 1 & 1 \end{array}\right]$ is the noise coefficient matrix. $w_{k-1}={\left[\begin{array}{ccc} w_{x\left(k-1\right)} & w_{y\left(k-1\right)} & w_{z\left(k-1\right)} \end{array}\right]}^{T}$ is the state noise.

### 3.2. Measurement Equation

In addition, the position of the robot is obtained by the MMW localization system. Because the localization system consists of noise interference, the position of the robot at k can be given as follows:

(23) $$\left[\begin{array}{c} x_{\mathrm{mR}(k)}\\ y_{\mathrm{mR}(k)}\\ z_{\mathrm{mR}(k)} \end{array}\right]=\left[\begin{array}{ccc} 1 & 0 & 0\\ 0 & 1 & 0\\ 0 & 0 & 1 \end{array}\right]\left[\begin{array}{c} x_{R\left(k\right)}\\ y_{R\left(k\right)}\\ z_{R\left(k\right)} \end{array}\right]+\left[\begin{array}{c} \upsilon_{x(k)}\\ \upsilon_{y(k)}\\ \upsilon_{z(k)} \end{array}\right]$$

where ${\left[\begin{array}{ccc} x_{\mathrm{mR}(k)} & y_{\mathrm{mR}(k)} & z_{\mathrm{mR}(k)} \end{array}\right]}^{T}$ is the only observed quantity. Therefore, the measurement equation of the system is:

(24) $$Z_{k}=HR_{k}+\upsilon_{k}$$

where $H=\mathrm{diag}\left[\begin{array}{ccc} 1 & 1 & 1 \end{array}\right]$ is the measurement matrix, and $\upsilon_{k}={\left[\begin{array}{ccc} \upsilon_{x(k)} & \upsilon_{y(k)} & \upsilon_{z(k)} \end{array}\right]}^{T}$ is the measurement noise.

### 3.3. Noise

In Equation (20), $w_{k-1}$ is related to the noise of the accelerometer. Without considering environmental vibration and large temperature changes, the noise of the accelerometer can be expressed as [27]:

(25) $$\delta a=\Delta_{a}+C_{a}+\eta_{a}$$

where $\Delta_{a}$ represents the zero drift of the accelerometer, $C_{a}$ represents the fixed error caused by the installation of the accelerometer and other factors, and $\eta_{a}$ represents the measurement noise of the accelerometer, which is Gaussian white noise. The compensation method is usually used to approximately eliminate $\Delta_{a}$ and $C_{a}$ In this case, the noise of the accelerometer is mainly random white noise and can be derived as follows:

(26) $$a_{n}\approx \eta_{a}$$

Therefore, $w_{k-1}$ in Equation (20) is an approximate white noise variable, and its mathematical expectation is as follows:

(27) $$E(w_{x\left(k-1\right)})=E(w_{x\left(k-1\right)})=E(w_{x\left(k-1\right)})\approx 0$$

In Equation (25), E represents the mathematical expectation of random variables; the variance matrix $w_{k-1}$ of $Q_{k-1}$ is:

(28) $$Q_{k-1}=\left[\begin{array}{ccc} D(w_{x(k-1)}) & 0 & 0\\ 0 & D(w_{y(k-1)}) & 0\\ 0 & 0 & D(w_{z(k-1)}) \end{array}\right]$$

where D represents the variance of random variables.

In Equation (22), $\upsilon_{k}$ is the measurement error of the MMW localization system. It has the characteristics of Gaussian white noise [16], and its mathematical expectation is as follows:

(29) $$E(\upsilon_{x(k)})=E(\upsilon_{y(k)})=E(\upsilon_{z(k)})=0$$

The measurement noise covariance matrix $G_{k}$ of the system is as follows:

(30) $$G_{k}=\left[\begin{array}{ccc} D(\upsilon_{x(k)}) & 0 & 0\\ 0 & D(\upsilon_{y(k)}) & 0\\ 0 & 0 & D(\upsilon_{z(k)}) \end{array}\right]$$

### 3.4. Filtering Algorithm

**Figure 3:**
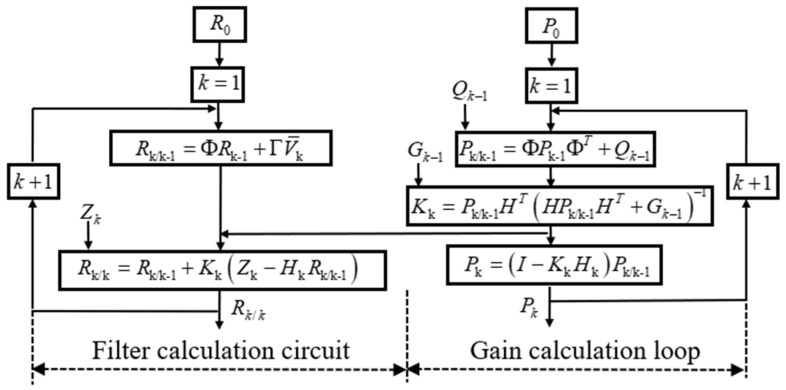
Integrated localization filtering algorithm and block diagram.

The measurement equation is established according to the millimeter wave localization system, the state equation is established according to the motion state of the system, and the position information of the robot is fused using the Kalman filter algorithm. The algorithm and information interaction are shown in Figure 3.

The algorithm proceeds as follows:

Step 1: At k, the predicted position coordinate $R_{k/k-1}$ of the robot can be deduced as follows: $R_{k/k-1}=\Phi R_{k-1}+\Gamma {\bar{V}}_{k}$.
Step 2: The mean square error of the estimate is: $P_{k/k-1}=\Phi P_{k-1}\Phi^{T}+Q$ ; $P_{k-1}$ is the best estimation error covariance at k − 1.
Step 3: The Kalman filter gain can be obtained: $K_{k}=P_{k/k-1}H^{T}{\left(HP_{k/k-1}H^{T}+G\right)}^{-1}$.
Step 4: The optimal estimation position coordinate of the robot can be obtained as follows: ${R_{k/}}_{k}=R_{k/k-1}+K_{k}\left(Z_{k}-H_{k}R_{k/k-1}\right)$.
Step 5: The mean square error between the optimal estimated position coordinate and the true position coordinate is given by $P_{k}=\left(I-K_{k}H_{k}\right)P_{k/k-1}$.

## 4. Experiment and Analysis

To verify the effectiveness of the proposed fusion localization system based on MMW radar and the INS, an experimental device using the system is designed.

### 4.1. Experimental Equipment

**Figure 4:**
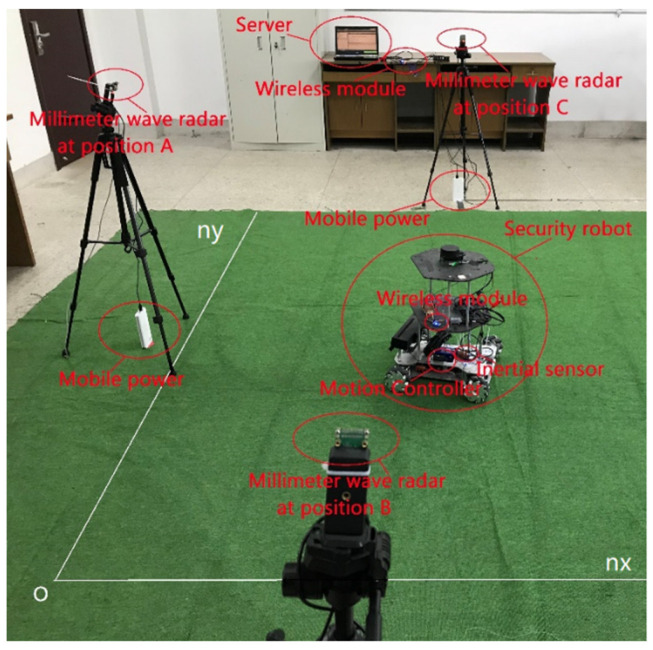
Construction and experimental setup of the safety simulation robot platform based on the fusion of the millimeter wave radar and the inertial localization system. The carrier in the figure is an omnidirectional (mecanum) four-wheel robot.

Experimental setup of the proposed fusion localization system is shown in Figure 4.

In Figure 4, the localization coordinate system $O_{n}X_{n}Y_{n}Z_{n}$ is constructed on a green carpet. The X, Y, and Z axes are orthogonal to each other at point O. Three MMV radars A, B, and C are placed on three tripods, and their position coordinates are (−1, 6, 1), (4, −1, 1), and (5, 11, 2), respectively. Three high-speed data differential signal lines with USB 3.0 are connected between the radars and a server, and communication between the server and the robot is achieved by a wireless module. The motion controller of the robot is an STM32 development board.

**Figure 5:**
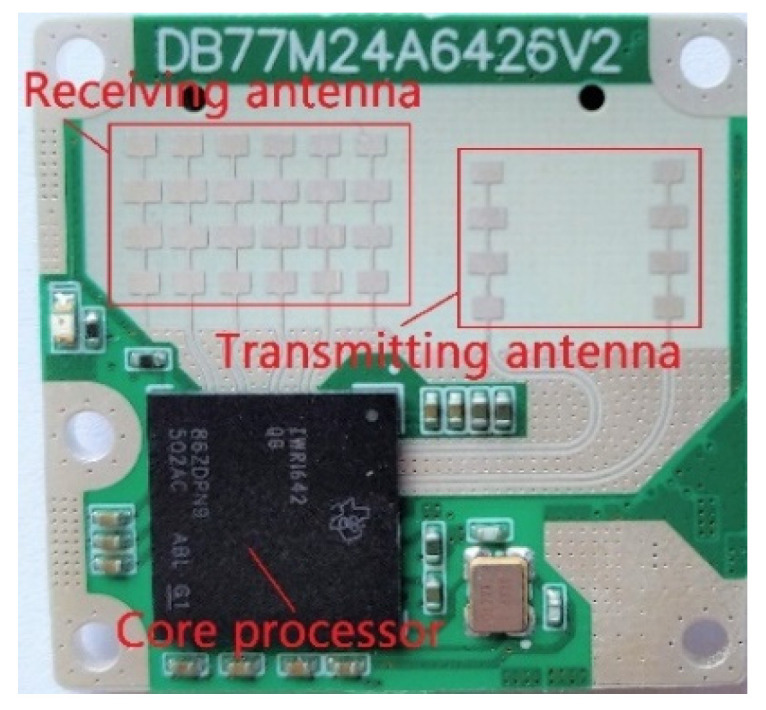
Model of an IWR1642 millimeter wave radar along with the position of the chip and antenna.

In the experimental scheme, IWR1642 radars are used in the MMW localization system; each radar has a working frequency band of 77–81 GHz, as shown in Figure 5.

**Figure 6:**
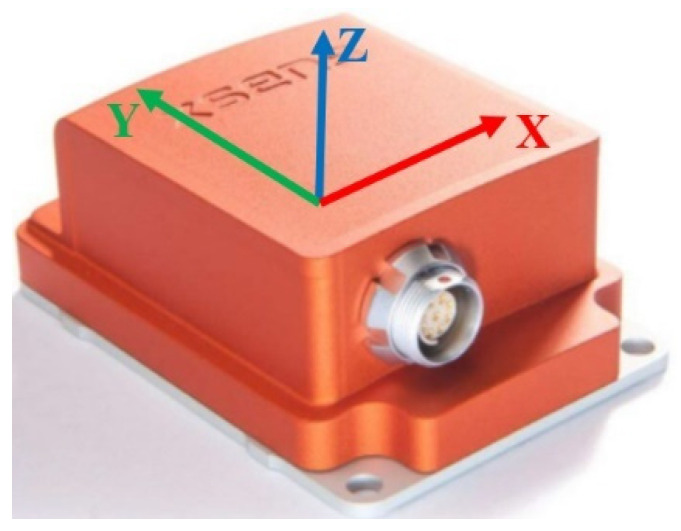
Reference position of the MTi-30 inertial localization system.

An MTi-30 strapdown inertial sensor is used for the INS, as shown in Figure 6.

**Figure 7:**
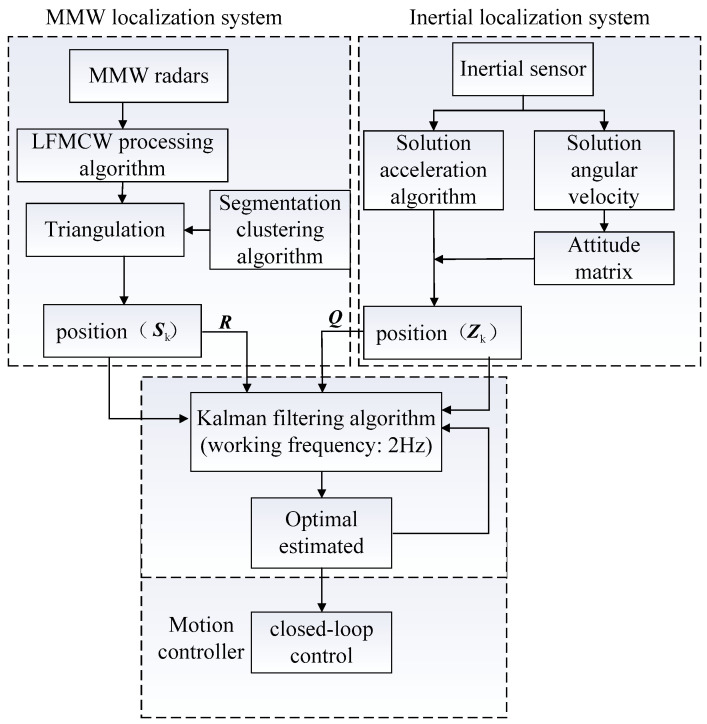
Information transmission and processing of the fusion localization system.

In the design scheme, the transmission and processing of information are as shown in Figure 7.

According to the figure, the robot position is output by the MMW radar and the INS. However, the output signal contains noise; therefore, the Kalman filter algorithm is used in the fusion localization system to estimate the optimal position of the robot. Then, this information is transmitted to the motion controller. Closed-loop control is performed in the controller according to current and the target position coordinate.

### 4.2. Experimental Parameters

#### Parameters of the MMW Localization System

The elevation angle of the radar is set to 10°. The radar’s minimum signal-to-noise ratio threshold is set to 45. The radar communication baud rate is set to 921,600. The range of the radar’s horizontal opening angle is −60° to 60°, and the range of the pitching angle is −19° to 19°. The noise covariance matrix of the MMW localization system is:

(31) $$G_{k}=\mathrm{diag}[(\begin{array}{ccc} 11\mathrm{cm}{)}^{2} & (11\mathrm{cm}{)}^{2} & 0 \end{array}]$$

(1) Parameters of the INS:

The standard full range of the gyroscope is 450°/s, and the bias stability in operation is 10°/h. The measurement range of accelerometer is 200 m/s^2^, and the bias stability in operation is 15 µg. MTi-30 outputs the attitude angle and velocity variation. In the experiment, the zero drift of the MTi-30 accelerometer was collected by making MTi-30 static for 10 min; $\Delta_{a}$ was then compensated. Based on the zero bias stability characteristics of gyroscopes and accelerometers, an INS error model is established to obtain the value of Q.

(2) Parameters of the fusion localization system:

Control period of the system: The two systems are fused per 0.5 s. The initial position is $R_{0}={\left[\begin{array}{ccc} 0 & 0 & 0 \end{array}\right]}^{T}$. The initial covariance matrix is set to $P_{0}={\left[0\right]}_{3\times 3}$.

(3) Parameters of robot motion control:

The length, width, and height of the four-wheel driving mobile robot are 45, 42, and 48 cm, respectively. The total mass is approximately 5.8 kg, and the maximum movement speed is approximately 1.2 m/s. The four motors are MD36N planetary gear motors. The software platform of the robot is the Free-RTOS operating system.

**Figure 8:**
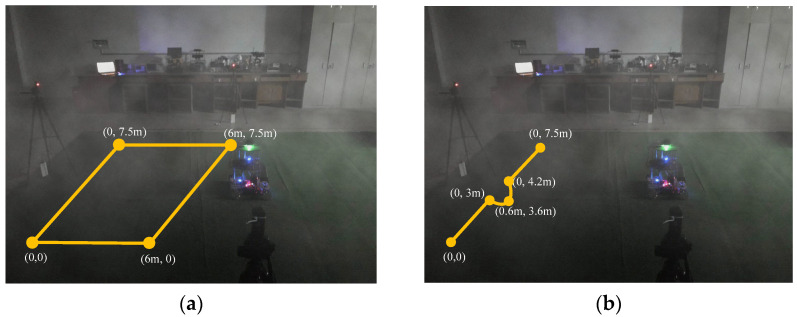
Experimental scene with smoke; (**a**) closed trajectory, (**b**) straight trajectory and an arc trajectory.

(4) Environment parameters:

Smog is created using cigarette cakes; it is mainly composed of ammonium chloride, flour, and rosin. The indoor light is insufficient, as shown in Figure 8.

The security robot is set to perform two tasks in Figure 8. The first task is to start from the starting point (0, 0) and run along a rectangle to test the localization effect of the robot when running along a closed trajectory, which is shown in Figure 8a; the starting point of another task is (0, 0) and the endpoint is (0, 7.5 m). During the operation, the robot needs to run along a 0.6 m radius arc to avoid obstacles, thus testing the localization effect of the robot when running between a straight trajectory and an arc trajectory, which is shown in Figure 8b.

### 4.3. Experimental Results and Analysis

In order to verify the effectiveness of the proposed algorithm, a test experiment of the approximate center method based on sparse point clouds was conducted first.

#### 4.3.1. Measurement of Robot Geometric Center

**Figure 9:**
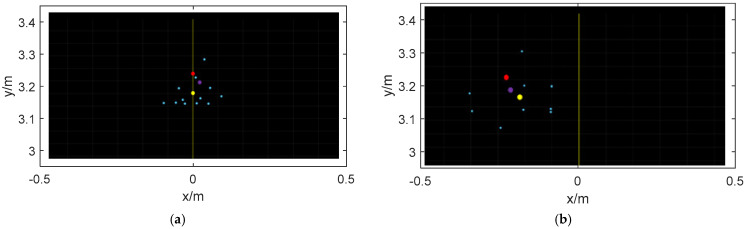
Radar point cloud distribution and processed approximate geometric center point distribution. (**a**) Experimental result 1. (**b**) Experimental result 2.

In the experiment, the environment first uses a radar to measure the geometric center of the robot, and compares the two methods. The two methods are direct mean processing and approximate center method for sparse point clouds. The geometric center of the robot is measured with a 1 mm accuracy scale and calculated using mathematical formulas. The experimental results are shown in Figure 9.

The blue dots in the image are the point clouds projected by the robot under the millimeter wave coordinate system after being scanned by the millimeter wave radar. The red dot is the reference geometric center point of the robot. The yellow point is the geometric center point obtained by directly processing the mean value of the blue point, and the purple point is the approximate geometric center point of the robot obtained by using the approximate center method of sparse point clouds.

According to Figure 9, it can be intuitively found that the center method based on sparse point clouds is closer to the geometric center of the robot than the direct mean method.

According to the above experimental parameters and methods, experiments were conducted on the fusion localization of the robot based on the MMW radar and inertial localization.

#### 4.3.2. Experimental Results for Robot Running Along Closed Trajectories

**Figure 10:**
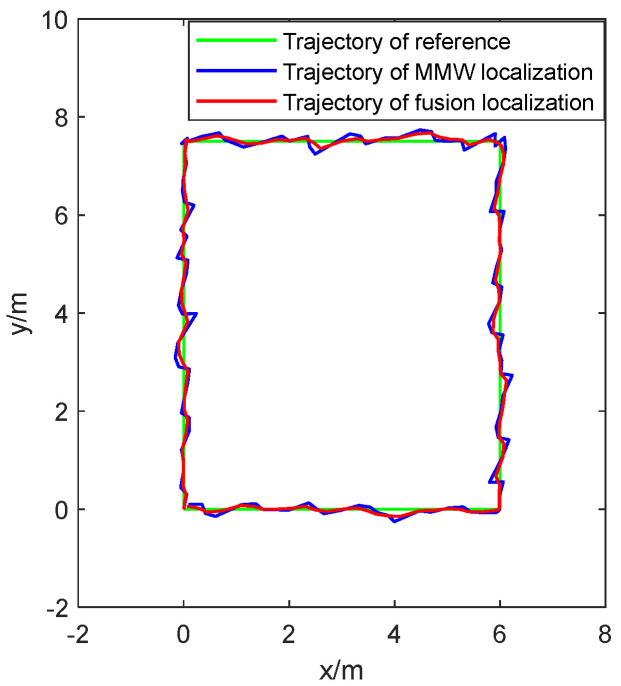
The localization effect of robots running along closed trajectories.

Running along the trajectory set in Figure 8a, the localization effect of the robot is shown in Figure 10.

In Figure 10, the green line represents the theoretical trajectory of the robot. The blue line represents the trajectory of the robot using MMW localization. The red line represents the trajectory of the robot using fusion localization. From the figure, it can be seen that the robot can run along a closed trajectory. Using fusion localization, the trajectory of the robot is closer to theoretical trajectory than using MMW localization.

**Figure 11:**
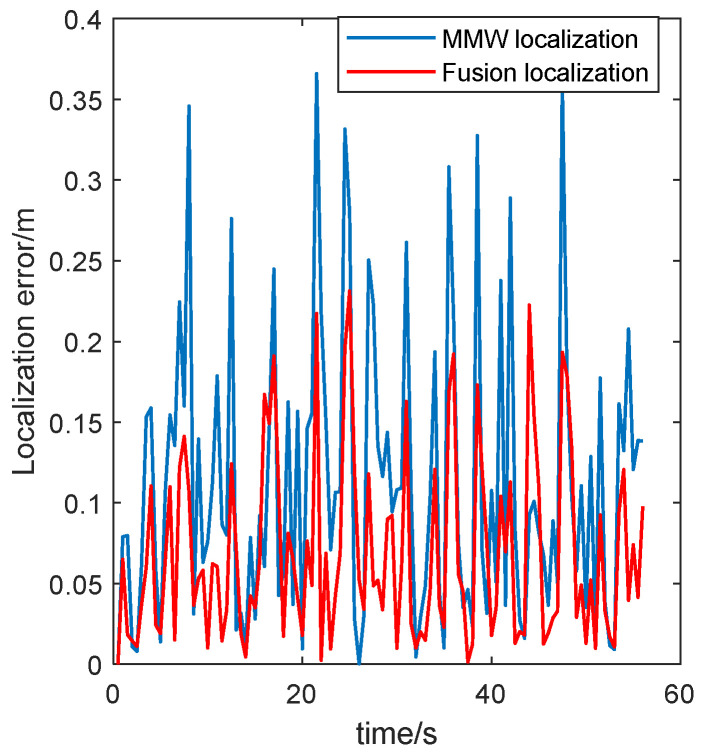
Localization error of robots running along closed trajectories.

To further compare the effect of the two localization methods, the localization error is shown in Figure 11.

As shown in Figure 11, The localization error was close to the error of the localization system with only MMW localization in the initial stage. After several iterations, the error of the fusion localization system was significantly reduced. The minimum error of MMW localization is 0.2 cm, the maximum error is 43.7 cm, and the average error is 11.5 cm. The minimum error of fusion localization is 0.1 cm, the maximum error is 23.1 cm, and the average error is 7.5 cm. This reflects a reduction of 34.8.0% in comparison to the MMW localization system.

**Figure 12:**
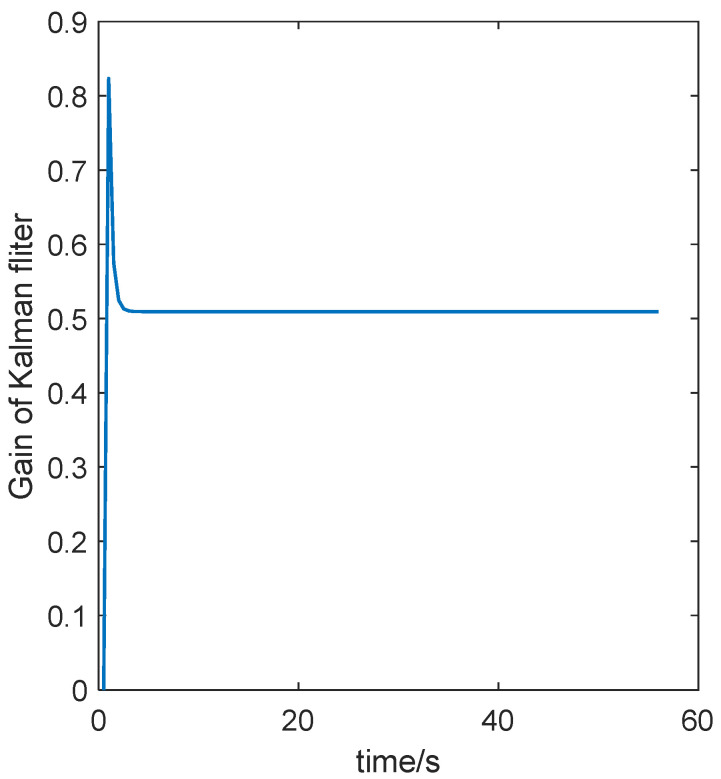
The gain of the fusion system.

For the results shown in Figure 10 and Figure 11, the gain of the fusion system is shown in Figure 12.

According to Figure 12, the initial gain is 0, followed by a gain reach of 0.778. After several sampling iterations, the gain stabilizes at 0.631, and the Kalman filter reaches a stable filtering state.

#### 4.3.3. Experimental Results for Robot Running Along Straight and Arc Trajectories

**Figure 13:**
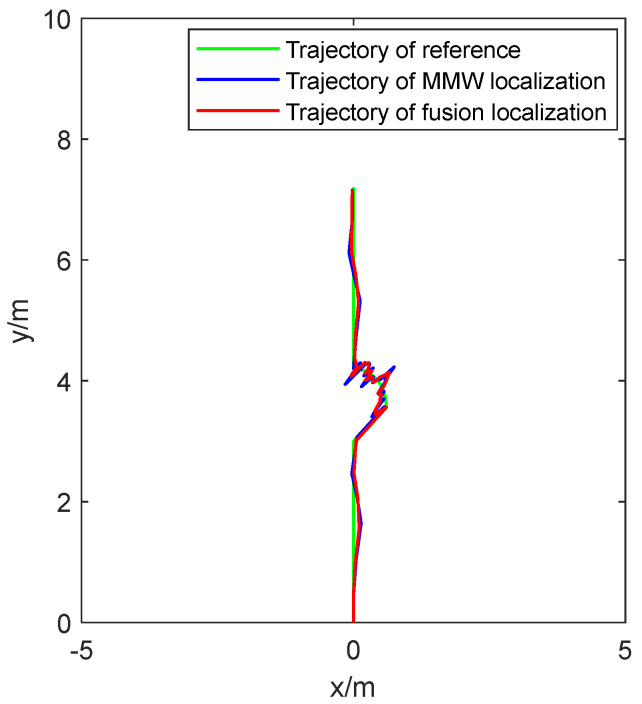
The localization effect of robots running along straight and arc trajectories.

Running along the trajectory set in Figure 8b, the localization effect of the robot is shown in Figure 13.

In Figure 13, the fusion localization system can effectively locate the robot when it runs between straight and arc trajectories; using fusion localization, the trajectory of the robot is closer to theoretical trajectory than using MMW localization. From Figure 13, it can also be seen that when the robot runs along an arc trajectory, the localization error of the robot is significantly larger than when it runs along a straight trajectory. This is mainly due to the increased error output by the inertial sensor during the robot’s curved motion.

**Figure 14:**
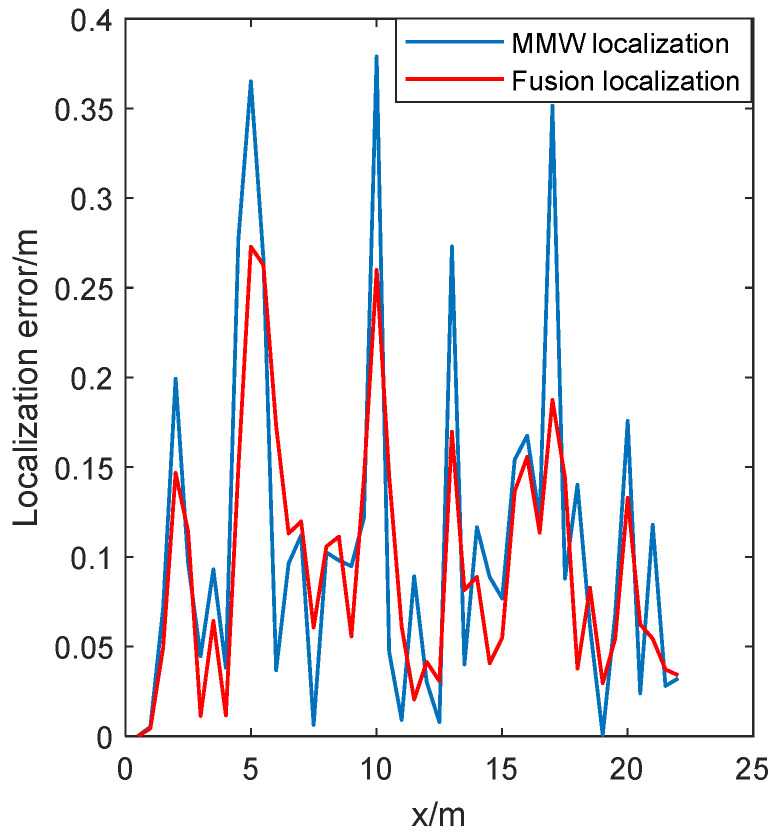
Localization error of robots running along straight and arc trajectories.

To further compare the effect of the two localization methods, the localization error is shown in Figure 14.

As shown in Figure 14, compared to running along the closed trajectory in the previous section, The localization error was closed to the error of the localization system with only the MMW radar in the initial stage. After several iterations, the error of the fusion localization system was significantly reduced. Due to the robot running along an arc trajectory from 3 s to 18 s, the average localization error increased slightly. In Figure 14, the minimum error of MMW localization is 0.2 cm, the maximum error is 39.8 cm, and the average error is 11.6 cm. The minimum error of fusion localization is 0.1 cm, the maximum error is 26.3 cm, and the average error is 8.3 cm. This reflects a reduction of 28.4% in comparison to the MMW localization system. Regarding the gain variation in the Kalman filter, as its trend is similar to Figure 12, it will not be shown here.

#### 4.3.4. Data Statistics from Experiments

For the closed trajectory shown in Figure 8a and the straight and arc trajectory shown in Figure 8b, 50 experiments were carried out separately. In order to verify the effectiveness of millimeter wave and inertial fusion localization systems in different environments, different lighting conditions (including good lighting and dim lighting) were selected in these experiments, and different smog concentrations were also chosen. The data obtained in the experiment are shown in Table 1.

From the data in the table, it can be seen that the error of the fusion localization system is smaller than that of the millimeter wave localization system; especially from the percentage reduction in average localization error, it can be seen that the reduction in localization error is significant. Therefore, the fusion localization system effectively improves the localization accuracy of the system and has good environmental adaptability.

## 5. Conclusions

To achieve autonomous operation of a robot in smog and dust environments and to improve its localization accuracy, a fusion localization system based on the MMW radar and INS was proposed. The experimental results confirmed that the localization error was close to the error of the localization system with only the MMW radar in the initial stage. After several iterations of the filtering algorithm, the gain matrix converged gradually and the error of the fusion localization system decreased, resulting in a stably operating robot. Compared to the localization system with only MMW radar, the average localization error is approximately reduced from 11 cm to 8 cm in the experiments, indicating that the fusion localization system has better localization accuracy. The difference between this method and satellite localization is that MMW uses signal frequency processing to calculate the distance between the radar and the robot, which avoids the need to consider the time differences between the transmitted signal and the received signal. Further, when more MMW radar devices are arranged, the robot can operate autonomously in a larger space. In addition, more filtering methods, such as particle filtering and neural network-based filters, can be used to reduce the error of the fusion localization system, which will also be carried out in the future. Finally, with the widespread application of pseudolite technology [28], the fusion localization system needs to be closely integrated with it to enhance the system’s ability to integrate communication and navigation, thus achieving smooth switching between outdoor and indoor localization for security robots.

## Figures and Tables

**Table 1:** Data statistics from experiments.

		The Average of Maximum Localization Error (cm)	The Average of Minimal Localization Error (cm)	The Average Localization Error (cm)	Percentage Reduction in Average Localization Error
closed trajectory	the MMW localization system	46.6	0.2	11.9	
	the fusion localization system	8.6	0.1	7.1	40.3%
straight and arc trajectory	the MMW localization system	45.6	0.2	11.7	
	the fusion localization system	10.9	0.1	8.8	24.8%

## Data Availability

The raw data supporting the conclusions of this article will be made available by the authors on request.
